# Translational AI and Deep Learning in Diagnostic Pathology

**DOI:** 10.3389/fmed.2019.00185

**Published:** 2019-10-01

**Authors:** Ahmed Serag, Adrian Ion-Margineanu, Hammad Qureshi, Ryan McMillan, Marie-Judith Saint Martin, Jim Diamond, Paul O'Reilly, Peter Hamilton

**Affiliations:** Life Sciences R&D Hub, Digital and Computational Pathology, Philips, Belfast, United Kingdom

**Keywords:** pathology, digital pathology, artificial intelligence, computational pathology, image analysis, neural network, deep learning, machine learning

## Abstract

There has been an exponential growth in the application of AI in health and in pathology. This is resulting in the innovation of deep learning technologies that are specifically aimed at cellular imaging and practical applications that could transform diagnostic pathology. This paper reviews the different approaches to deep learning in pathology, the public grand challenges that have driven this innovation and a range of emerging applications in pathology. The translation of AI into clinical practice will require applications to be embedded seamlessly within digital pathology workflows, driving an integrated approach to diagnostics and providing pathologists with new tools that accelerate workflow and improve diagnostic consistency and reduce errors. The clearance of digital pathology for primary diagnosis in the US by some manufacturers provides the platform on which to deliver practical AI. AI and computational pathology will continue to mature as researchers, clinicians, industry, regulatory organizations and patient advocacy groups work together to innovate and deliver new technologies to health care providers: technologies which are better, faster, cheaper, more precise, and safe.

## Introduction

Artificial Intelligence (AI) along with its sub-disciplines of Machine Learning (ML) and Deep Learning (DL) are emerging as key technologies in healthcare with the potential to change lives and improve patient outcomes in many areas of medicine. Healthcare AI projects in particular have attracted greater investment than in any other sector of the global economy (1). In 2018, it is estimated that $2.1 billion were invested in AI related products it is estimated this will rise to $36.1 billion dollars by 2025 (2).

The innovation opportunities offered by AI has been discussed extensively in the medical literature (3). Underpinned by the ability to learn from salient features from large volumes of healthcare data, an AI system can potentially assist clinicians by interpreting diagnostic, prognostic and therapeutic data from very large patient populations, providing real-time guidance on risk, clinical care options and outcome, but in addition provide up-to-date medical information from journals, textbooks, and clinical practices to inform proper patient care (4). By combining access to such extensive knowledge, an AI system can help to reduce diagnostic and therapeutic errors that are inevitable in conventional human clinical practice.

AI systems are being researched widely in healthcare applications, where they are being trained not just from one data modality but from multivariate data (5) generated across multiple clinical activities including imaging, genomics, diagnosis, treatment assignment where associations between subject features and outcomes can be learned. Big data is the ammunition for the development of AI applications. The increasing availability of enormous datasets, curated within and across healthcare organizations will drive the development of robust and generalizable AI apps in health. Currently the largest data sets come from diagnostic imaging (comprising CT, CAT, MRI, and MRA) and this tends to have been the focus of AI development in medicine.

The development of AI applications has been wide-raging. There are AI apps being researched and developed in health care from emergency call assessment of myocardial risk (6) to blood test analysis (7) to drug discovery (8). In parallel, FDA has been increasingly clearing AI medical applications for clinical use. These are summarized in Table 1.

**Table 1:** List of current FDA cleared AI applications for medical imaging.

**Company**	**Modality**	**Purpose**
IDx [IDx-DR]	Retinal image	Detection of diabetic retinopathy
MaxQ AI [Accipio Ix]	CT images	Prioritization of patients with
Imagen [OsteoDetect]	X-Ray images	Detection of wrist fractures
Zebra Medical Vision	CT images	Determination of cardiovascular disease risk
Arterys Inc. [Arterys Oncology AI suite, Arterys Cardio DL]	ARI, CT images	Expedited interpretation of images for detection of liver and lung cancer
	MRI	Segmentation of ventricles
Aidoc	CT images	Detection of intracranial hemorrhage
iCAD [ProFound AI^TM^]	Digital breast tomosynthesis	Cancer detection
Icometrix [icobrain]	MRI, CT	Quantification of clinical metrics for traumatic brain injury
Viz.ai	CT images	Detection of strokes
Subtle Medical [SubtlePET^TM^]	PET scans	Image enhancement

While, there is considerable promise in AI technologies in health, there are some challenges ahead. These include the ability of AI to generalize to achieve full automation in the diagnostic/clinical pathway will be extremely difficult. The medico-legal issues around accountability and liability in decision made or supported by machines will be hard, the regulatory issues for manufacturers of instruments capable of AI will be challenging and the need to demonstrate reproducibility and accuracy on large populations of patients which contain outliers and no-representative individuals may cause difficulties for AI development (9). Diagnosis and treatment plans are inherently non-linear, complex processes, requiring creativity, and problem-solving skills that demand complex interactions with multiple other medical disciplines. This will be difficult to achieve using AI. However, for well-defined domains that contribute to that diagnostic value chain, AI can clearly be transformative. Even with the advent of new AI, computers are unlikely to replace the diagnostic role of clinicians in the near future. However, there is a growing acceptance of AI systems with 61% of people suggesting that AI will make the world a better place (10).

Pathology is also now recognized as a strong candidate for AI development, principally in the field of cancer diagnosis and tissue biomarker analytics. This has been driven primarily by the development of whole slide imaging (WSI) platforms and digital pathology. Here, the generation high resolution digital images, each of which carries high volumes of data capturing the complex patterns, are critical to diagnosis of disease, providing a fertile opportunity to apply AI for improved detection of disease. This paper set out to review the recent applications of AI in pathology, highlighting the benefits and the pitfalls.

## Digital Pathology and AI: A Perfect Storm

With the advent of high throughput scanning devices and WSI systems, capable of digitally capturing the entire content of resection, biopsy and cytological preparations from glass slides at diagnostic resolution, researchers can now use these content rich digital assets to develop imaging tools for discovery and diagnosis. The advantages on quantitative pathology imaging have been known for many years. By extracting quantitative data from the images using automated segmentation and pixel analysis, diagnostic patterns and visual clues can be better defined driving improved reproducibility and consistency in diagnostic classification. Image analysis also allows the identification of sub-visual clues allowing the potential identification of new signatures of disease, derived from the pixel information, but not visible to the naked eye.

The advances in high throughput scanning devices in pathology has been astounding. In 2017, FDA cleared the use of the first WSI system for primary diagnostics (11). Here, digitization of pathology can enable pathologists to transform their entire workflow in a busy diagnostic laboratory; integrating digital scanners with laboratory IT systems, handling and dispatching digital slides to pathology staff inside and outside an organization, manually reviewing digital slides on-screen rather than using a microscope and reporting cases in an entirely digital workspace. This has been shown in the largest pivotal trial of digital pathology in the US to be non-inferior to conventional diagnosis by microscopy (12). With the right infrastructure and implementation, this has been shown to introduce significant savings in pathologists time in busy AP laboratories (13).

The digital transformation of pathology is expected to growth dramatically over the next few with increasing numbers of laboratories moving to high throughput digital scanning to support diagnostic practice. The real drivers for this include (i) an acute shortage of pathologists in many countries (14, 15), (ii) aging populations driving up pathology workloads (16), (iii) increased cancer screening programs resulting in increased workloads, (iv) increasing complexity of pathology tests driving up the time taken per case, (v) the need for pathology laboratories to outsource expertise (15, 16).

These same drivers are also accelerating the development of AI to support the diagnostic challenges that face pathologists today. By layering AI applications into digital workflows, potential additional improvements in efficiencies can be achieved both in terms of turn-around times but also patient outcomes though improved detection and reproducibility.

Recent reports from a number of professional pathology organizations have highlighted the potential that digital pathology and AI could bring to the discipline to address the current workforce, workload, and complexity challenges (16). The number of academic publications in pathology AI has increased exponentially with over 1,000 registered in PubMed in 2018. In the last 18 months there has been in excess of $100M invested in start-ups in pathology AI with a focus on building practical AI applications for diagnostics. In addition, governments are recognizing the opportunity that AI can bring to pathology. In the UK, a £1.3 billion investment has been announced to help detect diseases earlier through the use of artificial intelligence as part of the government's second Life Sciences Sector Deal (17). Pathology AI has been highlighted as a specific opportunity in UK and now $65M of investment has been committed to pathology and radiology AI R&D through a major Innovate UK initiative which has engaged industry and clinical sites across the UK (18).

This exciting and growing ecosystem of AI development in pathology is expected to drive major improvements in pathology AI over the next few years. This will require continued innovation in AI technologies and their effective application on large annotated image data lakes as develop in tandem with the adoption of digital pathology in diagnostic labs worldwide.

The convergence of advanced technologies, regulatory approval for digital pathology, digital transformation of pathology, adoption of digital pathology diagnostic practice, AI innovation and funding to accelerate pathology AI discovery, represents a perfect storm for the real transformation of pathology as a discipline.

## Deep Learning Methods in Pathology: A Rapidly Developing Domain

Given the widespread application of Artificial Intelligence (AI) based methods in computational pathology as illustrated in the previous section, it is worthwhile considering the current State of the Art in Deep Learning and the potential evolution of the technologies in the future. Although many of these are developed and proved in areas other than computational pathology, or indeed biomedical imaging, the field is moving forward apace, and many potential improvements will also have the capability of being used within computational pathology.

### Network Architectures

**Figure 1:**
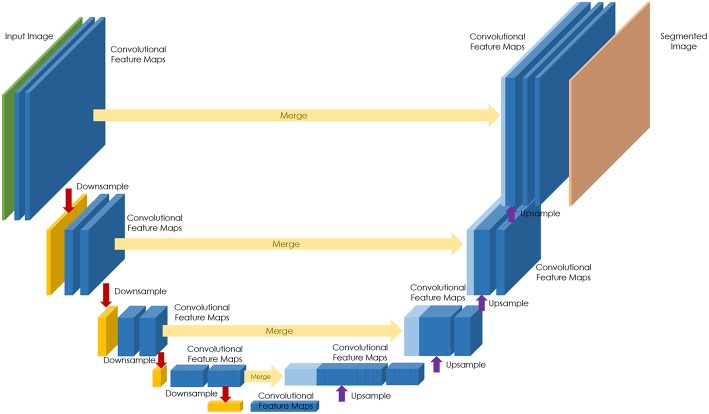
U-Net architecture for semantic segmentation, comprising encoder (downsampling), and decoder (upsampling) sections, and showing the skip connections between layers (in yellow).

The majority of efforts to date have focused on the development of neural network architectures in order to enhance the performance of different computational pathology tasks. U-Net has been commonly used in several applications (19–22). It relies on the strong use of data augmentation to use the available annotated samples more efficiently (Figure 1). Therefore, it became popular as it can be trained end-to-end from very few images, and, nevertheless, outperformed prior methods (based on a sliding-window convolutional network) on the ISBI challenge for segmentation of neuronal structures in electron microscopic stacks.

Recently, a deep learning network, called MVPNet, used multiple viewing paths for magnification invariant diagnosis in breast cancer (23). MVPNet has significantly fewer parameters than standard deep learning models with comparable performance and it combines and processes local and global features simultaneously for effective diagnosis. A ResNet based deep learning network (101-layer deep) was adopted in another work due to the fact of high efficiency and stable network structure (24). The method proved useful in discriminating breast cancer metastases with different pathologic stages from digital breast histopathological images.

A hybrid model was proposed for breast cancer classification from histopathological images (25). The model combines the strength of several convolutional neural networks (CNN) (i.e., Inception, Residual, and Recurrent networks). The final model provided superior performance compared against existing approaches for breast cancer recognition.

Motivated by the zoom-in operation of a pathologist using a digital microscope, RAZN (Reinforced Auto-Zoom Net) learns a policy network to decide whether zooming is required in a given region of interest (26). Because the zoom-in action is selective, RAZN is robust to unbalanced and noisy ground truth labels and can efficiently reduce overfitting. RAZN outperformed both single-scale and multiscale baseline approaches, achieving better accuracy at low inference cost.

### Generative Adversarial Networks

**Figure 2:**
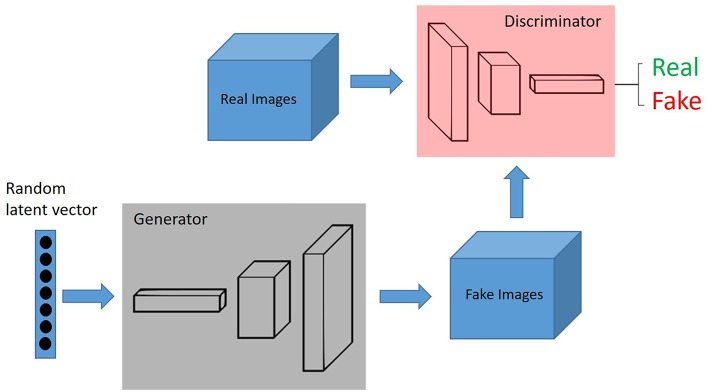
GANs, Generative adversarial networks (GANs) are deep neural network architectures comprised of two networks (generator and discriminator), opposing one against the other (thus the “adversarial”). The generator takes in random numbers and returns an image. This generated image is fed into the discriminator alongside a stream of images taken from the actual, ground-truth dataset.

Generative adversarial networks (GANs) are deep neural network architectures comprised of two networks (generator and discriminator), opposing one against the other (thus the “adversarial”) (Figure 2). GANs were introduced by Ian Goodfellow in 2014 (27), and has found its way for several applications in pathology.

For instance, color variations due to various factors are imposing obstacles to the digitized histological diagnosis process. Shaban et al. (28) proposed to overcome this problem by developing a stain normalization methodology based on CycleGAN, which is a GAN that uses two generators and two discriminators (29). They revealed that the method significantly outperformed the state of the art. Also, Lahiani et al. (30) used CycleGANs to virtually generate FAP-CK from Ki67-CD8 tissue stained images.

In order to create deep learning models that are robust to the typical color variations seen in staining of slides, another approach is to extensively augment the training data with respect to color variation to cause the models to learn color-invariant features (31). Recently, generative adversarial approaches (32, 33) have been proposed to learn to compose domain-specific transformations for data augmentation. By training a generative sequence model over the specified transformation functions using reinforcement learning in a GAN-like framework, the model is able to generate realistic transformed data points which are useful for data augmentation.

### Unsupervised Learning

Most deep learning methods require large annotated training datasets that are specific to a particular problem domain. Such large datasets are difficult to acquire for histopathology data where visual characteristics differ between different tissue types, besides the need for precise annotations.

Schlegl et al. (34) built an unsupervised learning to identify anomalies in imaging data as candidates for markers. The deep convolutional GAN learns a manifold of normal anatomical variability, accompanying a novel anomaly scoring scheme based on the mapping from image space to a latent space. Applied to new data, the model labels anomalies, and scores image patches indicating their fit into the learned distribution.

**Figure 3:**
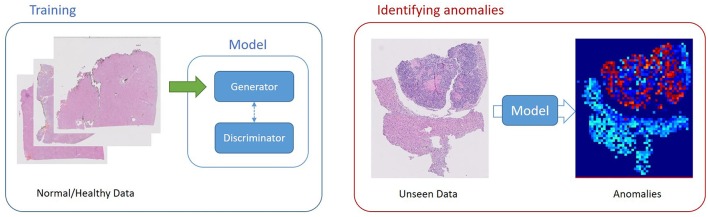
Unsupervised Learning, unsupervised anomaly detection framework. Generative adversarial training is performed on healthy data and testing is performed on unseen data.

In the context of domain adaptation, Xia et al. (35) proposed a new framework for the classification of histopathology data with limited training datasets. The approach utilizes CNNs to learn the low-level, shared image representations of the characteristics of tissues in histopathology images, and then optimizes this shared representation to a more specific tissue types (Figure 3).

The number, variation, and interoperability of deep learning networks will continue to grow as the field evolves. Pathology as a discipline and the technology available to apply deep learning modalities, must be able to adapt to these innovations to ensure the benefits on tissue imaging are fully experienced. This poses challenges in regulatory environment where algorithms need to be locked down to ensure evaluation, consistency, and repeatability and pace at which new algorithms can be taken to market. New approaches to regulatory governance need to be developed to ensure that patients benefit from the rapid deployment of latest technologies, but in a safe way. FDA and other regulatory authorities are exploring this with novel schemes that can accelerate new technologies to market (36).

## Testing the Ecosystem: Open Competitions in Computational Pathology and AI

One driving force behind innovation in computational pathology has been the increase of so-called “Grand Challenges.” These are open, public competitions aimed at addressing key use cases within the domain of computational pathology and typically provide data sets and annotations to allow competitors to develop algorithms, and test data and criteria against which those algorithms may be benchmarked and compared.

One of the earliest challenges in histopathology was held in 2010 at the International Conference for Pattern Recognition (ICPR) (37) which positioned two problems: (i) counting lymphocytes on images of H&E stained slides of breast cancer, and (ii) counting centroblasts on images of H&E stained slides of follicular lymphoma. These two problems are still pressing issues, as lymphocytic infiltration strongly correlates with breast cancer recurrence, and histological grading of follicular lymphoma is based on the number of centroblasts. Twenty-three groups registered for this challenge, but only five teams submitted their results with variable results.

The next grand challenge in histopathology was held in 2012 by the same ICPR conference group and focused only on mitosis detection in breast cancer histological images (38). Mitotic count is an important parameter in breast cancer grading. However, consistency, reproducibility, and agreement on mitotic count for the same slide can vary largely among pathologists. Detection of mitosis is a very challenging task since mitosis are small objects with a large variety of shape configurations. Different types of images were provided, so that the contestants could analyze classical images of H&E stained slides as well as images acquired with a 10 bands multispectral microscope, which might be more discriminating for the detection of mitosis. Compared to the previous ICPR challenge, 129 teams registered to the contest and 17 teams submitted their results, showing an increasing interest for automatic cell detection in general, and mitotic cell detection in particular. This was the first histopathology challenge where a deep learning max-pooling CNN clearly outperformed other methods based on handcrafted features, and paved the way for future use of CNNs (39).

The winner of the ICPR 2012 pathology grand challenge was also the winner of the following year's grand challenge Assessment of Mitosis Detection Algorithms 2013 (AMIDA13) held at the International Conference on Medical Image Computing and Computer Assisted Intervention (MICCAI 2013) (40). However, the AMIDA13 data set was much larger and more challenging than the one of ICPR 2012, with many ambiguous cases and frequently encountered problems such as imperfect slide staining. More than 89 research groups (universities and companies) registered, out of which 14 submitted results. The best performing method was the first system to achieve an accuracy that was in the order of inter-observer variability.

At the ICPR grand challenge in 2014, the objectives of the contest were to analyze breast cancer H&E stained biopsies in order to detect mitosis and also to evaluate the score of nuclear atypia (41). Nuclear atypia scoring is a value (1, 2, or 3) corresponding to a low, moderate or strong nuclear atypia respectively, and is an important factor in breast cancer grading, as it gives an indication about the aggressiveness of the cancer. The mitosis detection winning algorithm was a fast deep cascaded CNN composed of two different CNNs: a coarse retrieval model to identify potential mitosis candidates and a fine discrimination model (42). Compared with state-of-the-art methods on previous grand challenge data sets, the winning system achieved comparable or better results with roughly 60 times faster speed.

In 2015, the organizers of the International Symposium in Applied Bioimaging held a grand challenge (43) and presented a new H&E stained breast cancer biopsy dataset with the goal of automatic classification of histology images into one of four classes: normal tissue, benign lesion, *in situ* carcinoma, or invasive carcinoma. Once again, CNNs were highly successful (44) and achieved performance similar or superior to state of the art methods, even though a smaller and more challenging dataset was used. Main contributions of the winning system were image normalization based on optical density, patch augmentation and normalization, and training SVMs on features extracted by CNN.

MICCAI 2015 presented a new grand challenge in histopathology, on gland segmentation in H&E stained slides of colorectal adenocarcinoma biopsies, one of the most common form of colon cancer. An overview of the challenge along with evaluation results from top performing methods has been summarized (45). The same team that won the ICPR 2014 grand challenge also provided the winning CNN system for MICCAI 2015, but with fundamental differences between the systems. The novel deep contour-aware network (46) architecture consisted of two parts, a down-sampling path and an up-sampling path, resembling very much the well-known and popular U-Net architecture (20), which won the IEEE International Symposium on Biomedical Imaging (ISBI) cell tracking challenge in the same year and was also conditionally accepted and published at MICCAI 2015.

The organizers of MICCAI 2016 presented the TUmor Proliferation Assessment Challenge (TUPAC 2016) (47) with a very clear and testing objective: predicting mitotic scores (1, 2, or 3) of nuclear atypia in images of breast cancer H&E stained slides, one of the ICPR 2014 goals. However, the main difference from previous conferences was the fact that contestants had to analyze whole slides images (WSI) instead of regions of interest manually selected by pathologists. The challenge was based on a very large dataset called The Cancer Genome Atlas (TCGA) (48, 49) that also included genomic information, so the contestants had an additional objective of predicting PAM50 gene expression scores. The system that won both tasks (50) performed image preprocessing (tissue detection with Otsu thresholding and stain normalization) and ROI detection based on cell density, followed by feature extraction using a hard-negative mined ResNet (51) architecture, which they then used as input to an SVM.

The organizers of ISBI 2016 also presented a grand challenge based on WSI: the Cancer Metastases in Lymph Nodes Challenge 2016, or CAMELYON16 (52). The main objective was to assess the performance of automated deep learning algorithms at detecting metastases in H&E stained tissue sections of lymph nodes with breast cancer and compare it with diagnoses from (i) a panel of 11 pathologists with time constraint and (ii) one pathologist without any time constraint. Performance assessment was done on two main tasks, (i) metastasis identification and (ii) WSI classification as either containing or lacking metastases. The winning team submitted a CNN system that performed image preprocessing first (tissue detection and WSI normalization) and relied on a pre-trained 22-layer GoogLeNet architecture (53) to identify metastatic regions for the first task of the challenge. Afterwards they did post-processing and extracted features that were used to train a random forest classifier for the second task of the challenge. The winning system performed better than the panel of 11 pathologists with time control and had comparable results to the only one pathologist without any time control.

Building upon the success of CAMELYON16, ISBI 2017 introduced CAMELYON17 (53), the grand challenge with the largest histopathology dataset publicly made available, totaling 1399 WSI and around 3 terabytes (54). The main objective changed slightly, from individual WSI analysis to patient level analysis (i.e., combining the assessment of multiple lymph node slides into one outcome). The winning system came from the same team that won TUPAC 2016 and was based on an ensemble of three pre-trained ResNet-101, each of them further optimized with different patch augmentation techniques for the CAMELYON17 dataset.

ISBI 2017 also introduced a grand challenge for Tissue Microarray (TMA) analysis in thyroid cancer diagnosis (55). The main objective of this challenge was predicting clinical diagnosis results based on patient background information, but also on H&E stained TMAs as well as immunohistochemical (IHC) TMAs.

MICCAI 2018 presented three different challenges that used histopathology images from H&E stained biopsies. Two of them took place within the workshop for Computational Precision Medicine: (1) Combined Radiology and Pathology Brain Tumor Classification and (2) Digital Pathology Nuclei Segmentation. The first one focused on classifying low-grade from high-grade brain tumors based on a combination of radiology and pathology images, while the second one focused on nuclei segmentation in pathology images acquired from low-grade and high-grade brain tumors. The third challenge was the Multi-organ nuclei segmentation (MoNuSeg) challenge and was based on a public dataset (56) containing 30 images and around 22,000 nuclear boundary annotations from multiple organs.

In 2018, the widely-used and popular competitions website Kaggle opened submission for the Data Science Bowl with the main objective of segmenting nuclei on microscopy images acquired under different conditions and from different organs. The dataset included both H&E stained biopsies as well as fluorescence images. The winning system selected from over more than 700 submissions was an ensemble of four very deep CNNs, trained using heavy augmentation techniques, and a complex post-processing step involving water-shedding, extracting morphological features and training gradient boosted trees.

Another challenge that took place in 2018 was the Grand Challenge on BreAst Cancer Histology (BACH) (57), held at the International Conference on Image Analysis and Recognition (ICIAR 2018). This challenge was a follow-up challenge of Bioimaging 2015, and the purpose was classification at the slide-level and pixel-level of H&E stained breast histology images in four classes: normal, benign, *in situ* carcinoma and invasive carcinoma. The winning system of both tasks (58) was based on the Inception-ResNet-v2 architecture (59), improved by a modified hard negative mining technique.

Going forward in 2019, at least three challenges have been announced, showing the massive interest that exists in the online communities for solving complex pathology problems. Kaggle's Data Science Bowl 2019 aims at identifying metastatic tissue in histopathologic scans of lymph node sections, building on the huge success and massive dataset of the CAMELYON challenges. The 2019 SPIE Medical Imaging Conference will hold the BreastPathQ challenge, with the main purpose of quantifying tumor patch cellularity from WSI of breast cancer H&E stained slides. ISBI 2019 will also hold another challenge in Automatic Cancer Detection and Classification in Whole-slide Lung Histopathology.

## Selected Applications of AI in Pathology

### Prostate Cancer

Prostate cancer is the second most common cancer in men in USA and the most common cause of cancer death in men in the UK, with around 175,000 new cases per year in the US (60), 47,200 new cases per year in UK (61) with 9.6 million deaths globally from the disease. Histopathological assessments, using needle core biopsies and surgical resection, play an important role in the diagnosis of the prostate cancer. Current interpretation of the histopathology images includes the detection of tumor patterns, Gleason grading (62), and the combination of prominent grades into a Gleason score, which is critical in determining the clinical outcome. The higher the Gleason grade and the more prominent that pattern is seen in biopsies, the more aggressive the cancer the more likely that that disease has already metastasized. Gleason grading is not only time-consuming, but also prone to intra- and inter-observer variation (63, 64). Tissue and cellular imaging have for a long time been proposed as a quantitative tool in the assessment of cancer grade in the prostate. However, this has been limited by the technology and the precision of the imaging algorithms. More recently, several research teams have proposed to use AI technologies for the automated analysis of prostate cancer as a means to precisely detect prostate cancer patterns in tissue sections and also to objectively grade the disease.

With regard to tumor detection in prostate tissues, Litjens et al. (65) used a convolutional auto-encoder for tumor detection in H&E stained biopsy specimens. Substantial gains in efficiency were possible by using CNNs to exclude tumor-negative slides from further human analysis; showing the potential to reduce the workload for pathologists. Bulten et al. (66) developed an algorithm for automated segmentation of epithelial tissue in prostatectomy slides using CNN. The generated segmentation can be used to highlight regions of interest for pathologists and to improve cancer annotations.

While tumor detection is largely a binary decision on the presence or absence of invasive cancer in tissue biopsies, Gleason grading represents a complex gradation of patterns that reflect the differentiation and so the severity of the cancer. Such is the complexity of the image patterns seen, reliable and consistent interpretation is challenging and prone to disagreement and potential diagnostic error. Jiménez-del-Toro et al. developed an automated approach using patch selection and CNN, to detect regions-of-interest in WSIs where relevant visual information can be sampled to detect high-grade Gleason grades (67). They achieved an accuracy of 78% on an unseen data set, with particular success in classifying Gleason Grades 7–8.

A number of groups have used a generically trained CNN for analyzing prostate biopsies and classifying the images into benign tissue and different Gleason grades (68, 69). The proposed algorithm benefited from combining visually driven feature extraction by human eye with those derived by a deep neural network (69). Importantly, this work showed was able to differentiate between Gleason 3+4 and 4+3 slides with 75% accuracy. The algorithm was designed to run on whole slide images, conceptually allowing the technology to be used in clinical practice.

One group (70) presented a deep learning approach for automated Gleason grading of prostate cancer tissue microarrays with H&E staining. The study shows promising results regarding the applicability of deep learning based solutions toward more objective and reproducible prostate cancer grading, especially for cases with heterogeneous Gleason patterns.

Nagpal et al. (71) presented a deep learning system for Gleason grading in whole-slide images of prostatectomies. The system goes beyond the current Gleason system to more finely characterize and quantitate tumor morphology, providing opportunities for refinement of the Gleason system itself. This approach opens the opportunity to build new approaches to tissue interpretation; not based on simply measuring what pathologists recognize in the tissue today, but that creates new signatures of disease that radically transform the approach to diagnosis and has stronger correlation with clinical outcome.

A compositional multi-instance learning approach has also been developed which encodes images of nuclei through a CNN, then predicts the presence of metastasis from sets of encoded nuclei (72). The system has ability to predict the risk of metastatic prostate cancer at diagnosis.

In conclusion, AI and deep learning techniques can play an important role in prostate cancer analysis, diagnosis and prognosis. They could also be used to quickly analyze huge clinical trial databases to extract relevant cases. Although, the above techniques have focused on the use of H&E stained images, techniques that use immunohistochemistry might be of more interest when researching the efficacy of drugs or the expression of genes.

### Metastasis Detection in Breast Cancer

The problem of identifying metastases in lymph nodes within the context of breast cancer is an important part of staging such cancers, but has been found to be a challenging task for pathologists, with one study showing a change of classification in up to 24% of cases after subsequent review (73). The applicability of AI-based techniques to assist pathologists with this task has been addressed by a number of open competitions such as the CAMELYON series discussed earlier (52–54, 59, 74), and the results from those challenges have shown comparable discriminative performance to pathologists, in the particular task of detecting lymph node metastases in H&E-stained tissue sections (52). There have been a number of subsequent studies in metastasis detection (31, 75, 76). The work by Liu et al. (31) is particularly interesting as it showed superiority for algorithm-assisted pathologist detection of metastases over detection by pathologist or algorithm in isolation. The potential benefits of AI in this use case are yet to be studied in a clinical trial, but the work of Benjordi et al. (52) and Liu et al. (31) indicates the potential for AI to assist pathologists in making difficult clinical decisions, and improve the quality and consistency of such decisions.

### Ki67 Scoring

The Ki67 antigen is a nuclear protein strictly associated with cell proliferation. This makes it a perfect cellular biomarker for determining the growth factor of any given cell population, which has particular value in cancer research, where cell proliferation is strong marker of tumor growth and patient prognosis. The fraction of Ki67 positive tumor cells (Ki-67 labeling index, i.e., Ki67 LI) is often correlated with the clinical course of the disease (77). In breast cancer research there has been a massive international multicenter collaboration toward the validation of a standard Ki67 scoring protocol (78–80) as well as showing the prognostic value of an automated Ki67 protocol compared to manual or visual scoring (81, 82). In prostate cancer research, Ki67 has been validated as a biomarker for overall survival (83) and disease free survival in a large meta-analysis (84), but a standard scoring process is still missing (85).

Some authors have show significant agreement between their automated Ki67 LI and the average of two pathologists KI67 LI estimates (86). Their model is based on image preprocessing (color space transformation), image clustering with k-means, and cell segmentation and counting using global thresholding, mathematical morphology and connected component analysis.

Some have chosen to analyze the cell counting task as a regression problem (87). They modified a very deep ResNet with 152 layers to output a spatial density prediction and evaluated it on three datasets, including a Ki67 stained dataset, compared their approach to three state-of-the-art models and obtain superior performance. The same authors were also the first to combine deep learning with compressed sensing for cell detection (88). The essential idea of their method was to employ random projections to encode the output space (cell segmentation masks) to a compressed vector of fixed dimension indicating the cell centers. Afterwards, the CNN regresses this compressed vector from the input pixels. They achieved the highest or at least top-3 performance in terms of F1-score, compared with other state-of-the-art methods on seven mainstream datasets, including the one from (87).

A novel deep learning technique based on hypercolumn descriptors of VGG16 for cell classification in Ki67 images has been proposed, called Simultaneous Detection and Cell Segmentation (DeepSDCS) (89). They extracted hypercolumn descriptors to form an activation vector from specific layers to capture features at different granularity. These features were then propagated using a stochastic gradient descent optimizer to yield the detection of the nuclei and the final cell segmentations. Subsequently, seeds generated from cell segmentation were propagated to a spatially constrained CNN for the classification of the cells into stromal, lymphocyte, Ki67-positive cancer cell, and Ki67-negative cancer cell. They validated its accuracy in the context of a large-scale clinical trial of estrogen-receptor-positive breast cancer. They achieved staggering accuracies of 99% and 89% on two separate test sets of Ki67 stained breast cancer dataset comprising biopsy and whole-slide images.

A model has been proposed for GEP-NEN based on three parts: (1) a robust cell counting and boundary delineation algorithm that is designed to localize both tumor and non-tumor cells, (2) online sparse dictionary learning method, and (3) an automated framework that is used to differentiate tumor from non-tumor cells and then immunopositive from immunonegative tumor cells (90). They report similar performance to pathologists' manual annotations.

Other authors have shown the improved performance of a modified CNN model over classical image processing methods for robust cell detection in GEP-NEN, testing their algorithm on 3 data sets, including Ki67 and H&E stained images (91, 92).

### Deep Learning for Immuno-Histochemistry Applications Including PD-L1

IHC image analysis provides an accurate means for quantitatively estimating disease related protein expression, thereby reducing inter- and intra-observer variation and improve scoring reproducibility. For this reason, accurate approximation of staining in IHC images for diagnostics has long been an important aspect of IHC-based computational pathology. Many commercial and open source solutions are available that allow IHC analysis evaluation for research and discovery purposes (25).

A variety of challenges exist in IHC analytics. Shariff et al. provide a review of the problems faced in the domain of IHC image analysis along with solutions and techniques used in the area (93). Subsequently several image processing and machine learning based approaches have been proposed providing different levels of accuracies (94–96). Then work of Lejeune et al. (97) is significant as they perform automated analysis for quantification of proteins for different nuclear (ki67, p53), cytoplasmic (TIA-1, CD68) and membrane markers (CD4, CD8, CD56, HLA-Dr). Techniques used involve extracting contrast features in combination with spatial filtering followed by color segmentation with the help of HSI histogram-based model. Range-based filtering is followed by automatic counting using measurement data and statistics (97). Similarly, Humphries et al. use image processing to detect stained tumor cells in order to understand the role of PD-L1 in predicting outcome of breast cancer treatment (98). Also, Zehntner et al. propose a novel technique for automatic image segmentation using the blue to red channel ratio and subsequently use different thresholds on B/R and the green channel to acquire chromogen positive areas. The results show high level of concordance with manual segmentation (99).

Recently, traditional image processing and machine learning techniques have been shown to be less powerful and efficient as compared to deep learning techniques (100–102). Chen and Chefd'Hotel propose a CNN-based technique for automatic detection of immune cells in IHC images. A very important aspect of the work is that sparse color un-mixing is used to preprocess the image in to different biologically meaningful color channels (103). Lahiani et al. trained a unified segmentation model with a color deconvolution segment added to the network architecture. High accuracy results are obtained with substantial improvement in generalization. The added deconvolution segment layer learns to differentiate stain channels for different types of stains (104). Garcia et al. used CNNs for detection of lymphocytes in IHC images and have used augmentation to increase the data for analysis. The technique works on single-cell as well as multiple-cell images (105).

**Figure 4:**
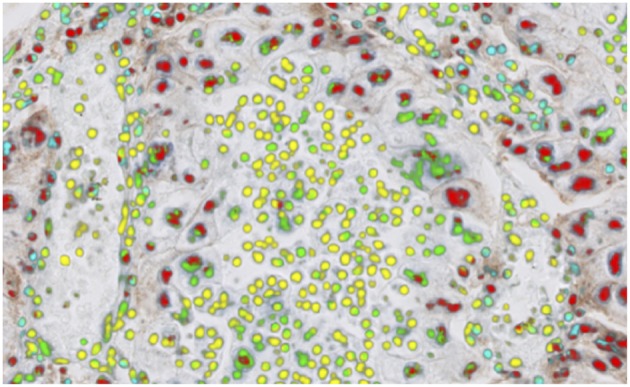
PD-L1 imaging in lung cancer. Deep learning can be used to identify and distinguish positive | negative tumor cells and positive | negative inflammatory cells.

One biomarker of recent interest has been PD-L1 expression, which is used as a companion or complementary marker to stratify patients who may benefit from checkpoint inhibitor therapy in a number of cancer types (106). The quantification of this biomarker is made more difficult by the non-specific staining of areas other than tumor cell membranes, in particular macrophages, lymphocytes, necrotic and stromal regions. These factors can, in particular cause challenges when scoring around the 1% threshold used for second-line treatment of NSCLC with pembrolizumab (107). Automated imaging based on deep learning of the cell types and the expression profiles can significantly underpin the quantitative interpretation of PD-L1 expression (Figure 4). The work by Humphries et al. (107) describes the use of image processing to quantify PD-L1 expression and showed reasonable concordance with scores from trained pathologists for adenocarcinoma and squamous cell carcinomas in lung. Another recent study (108), applied deep learning to determination of the PD-L1 Tumor Proportion Score (TPS) in NSCLC needle biopsies, showing strong concordance between the algorithmic estimation of TPS and pathologist visual scores.

### Genetic Mutation Prediction

Recently, some have used deep CNNs to predict whether or not SPOP was mutated in prostate cancer, given only the digital whole slide after standard H&E staining (109). Moreover, quantitative features learned from patient genetics and histology have been used for content-based image retrieval, finding similar patients for a given patient where the histology appears to share the same genetic driver of disease i.e., SPOP mutation, and finding similar patients for a given patient that does not have that driver mutation. This is extremely beneficial as mutations in SPOP lead to a type of prostate tumor thought to be involved in about 15% of all prostate cancers (110).

Within the same context, Coudray et al. (111) trained the network to predict the ten most commonly mutated genes in LUAD. Also, Kim et al. (112) used deep convolutional neural networks to predict the presence of mutated BRAF or NRAS in melanoma histopathology images. The findings from these studies suggest that deep learning models can assist pathologists in the detection of cancer subtype or gene mutations and therefore has the potential to become integrated into clinical decision making.

### Tumor Detection for Molecular Analysis

**Figure 5:**
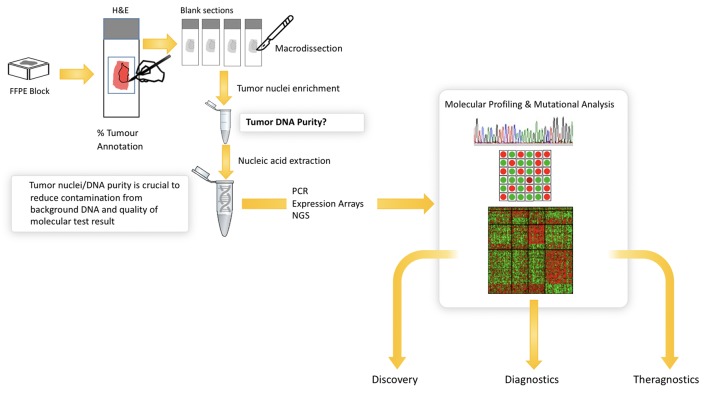
Illustrates the current workflow in molecular research and diagnostics. Solid tumor analysis is commonly derived from FFPE block and H&E tissue section as guide for tumor content (far left). The figure shows the need for annotation and macrodissection and the importance of tumor purity from FFPE samples for molecular profiling. Digital pathology can automate the annotation and measurement of tumor cells in H&E—providing a more objective, reliable platform for molecular pathology.

The increasing number of molecular tests for specific mutations in solid tumors has significantly improved our ability to identify new patient cohorts that can be selectively treated. EGFR mutational analysis in lung cancer, KRAS in colorectal cancer and BRAF in melanoma all represent examples of mutational tests that are routinely performed on appropriate patients with these cancers. Similarly, multigene panels are increasingly being used to better profile patients for targeted therapy, and next generation sequencing is routinely performed for solid tumor analytics and is now becoming the standard of care in many institutions. In all of these settings, histopathological review of the H&E tissue section prior to molecular analysis is critical (Figure 5). This is due to the heterogeneity that exists in most tissue samples where clarity over the cellular content is critical to ensuring the quality of the molecular test. Manual mark-up of the tumor in the tissue section is often carried out to support macrodissection, aimed at enriching the tumor DNA. Here, the molecular test is carried out on tumor tissue scraped from the FFPE, H&E tissue section. Also, given the heterogeneity of solid tumor tissue samples and the multiple tumor and non-tumor cells that exist in a sample, the pathologist must routinely assess the % of tumor cells to again ensure that there is sufficient tumor DNA in the assay and that the background noise from non-tumor cells does not impact on the test result. Most tests have a % tumor threshold below which the test is not recommended.

The challenge is that the interobserver variation in the assessment of percentage of tumor is considerable (113–116) where differences can range from between 20% and 80% and where the risk of false negative molecular tests, due to imprecise understanding of sample quality, could impact on patient care.

**Figure 6:**
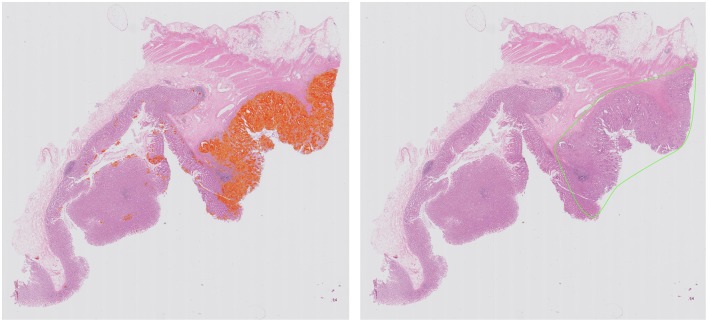
Automated identification of colorectal tumor in H&E tissue samples using deep learning networks, showing heatmap of tumor regions (**Left**) and automatically generated macrodissection boundary (**Right**) with a product called TissueMark^1^.

**Figure 7:**
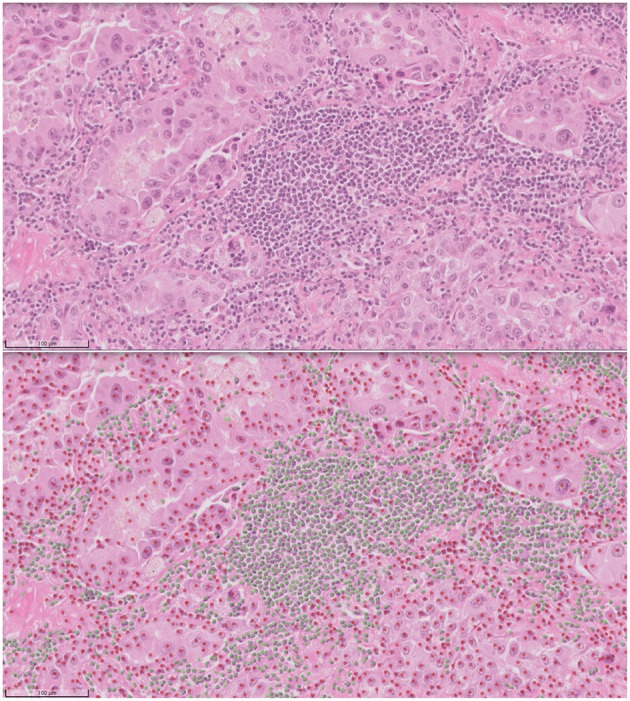
Automated analysis of cellular content in H&E using deep learning in TissueMark^1^. Here tumor (red) and non-tumor cells (green) can be distinguished, annotated for visual inspection and counted to reach more precise qualititive measures of % tumor across entire whole slide H&E scans in lung, colon, melanoma, breast, and prostate tissue sections.

Computational pathology and image analytics have been used to develop a solution for automated analysis and annotation of H&E tissue samples, identifying the boundary of the tumor and precisely measuring tissue cellularity and tumor cell content. *TissueMark*^1^ developed by PathXL Ltd and subsequently by Philips has been described in the literature (113). Designed specifically to support molecular pathology laboratories, it has been shown high levels of performance in lung cancer. The algorithms have now been expanded to automatically identify tumor in colorectal, melanoma, breast, and prostate tissue section. Trained on large datasets across multiple laboratories and sing deep learning technology, the solution can drive automation of microdissection and quantitative analysis of % tumor, providing an objective tissue quality evaluation for molecular pathology in solid tumors (Figures 6, 7).

## Challenges With Computational Pathology as a Diagnostic Tool

As can be seen from this review, there has been considerable research on AI and deep learning across many pathological problems. Indeed, a review in PubMed shows an almost exponential growth in publications in pathology AI on the last 5 years. Unfortunately, as is typical, this has not been mirrored by a similar growth in diagnostic practice and the translation of research to clinical diagnostics.

Some of the reasons for this are shown in Table 2. A key requirement for technology translation is the need to embed AI within diagnostic workflow—to ensure that the pathologist can easily access AI applications for diagnostics. With the first approval to use digital pathology for primary diagnostics in the US and increasing adoption of digital WSI scanners, image management systems and workflows in digital diagnostic practice, this presents the ideal platform on which to build AI applications. Here AI should be fully embedded seamlessly within diagnostic workflow, where the pathologist can review digital slides manually for conventional manual diagnostic assessment but also access new visual and quantitative data generated from computational pathology imaging. Computational should not represent an extra step, the need to load new software or a switch in context, but should practically invisible, operating in the background but generating the valuable insights into tissue analytics that are not currently available.

**Table 2:** List of the key challenges that face the translation of computational pathology into clinical practice.

**Key challenges in diagnostic AI in pathology**
Access to large well-annotated data sets Context switching between workflows Algorithms are slow to run Algorithms require configuration Properly defined protocols for training and evaluation Algorithms are not properly validated Lack of health economics

*These typically slow down access to diagnostic apps or make pathologists hesitant to adopt. It is critical to ensure that apps are embedded in digital workflows to allow seamless access to AI*.

In designing algorithms, it is critical to ensure that algorithms execute quickly to avoid slowing the diagnostic process. This requires optimal processing hardware to be in place to manage analytical requests made by the pathologist within the viewing software. Better still is to have the images completely analyzed at the time of scanning and to allow all of the relevant image analysis data to be available to pathologist at the time of review. This includes the use of computational pathology to dispatch digital slides to the correct pathologist, prioritize cases for review, and request extra sections/stains before pathological review. This requires considerable processing capacity available at the time of scanning with pixels being analyzed as they are created on the scanner. However, bringing intelligence to pathology workflow in in this way will potentially drive further efficiencies in pathology, accelerate turnaround times and improve the precision of diagnosis.

Finally, as stated previously, translation into clinical practice and adoption by pathologists requires algorithms trained and validated on large patient cohorts and sample numbers, across multiple laboratories. Many academic studies are restricted to small sample sets from a single laboratory. The preparation of pathology specimens has long been recognized as a problem which can challenge the robustness of computational pathology algorithms (106, 107), and this continues to be problem for the larger data sets required for deep learning. While approaches such as augmentation and/or color normalization have been used successfully in training such algorithms (98, 108), adequately representing inter-laboratory variations in the training data will also give confidence that algorithms are not “over-trained” to perform well on the characteristics of only one lab (preparation/staining). However, for the validation of such algorithms for wider usage, it is absolutely necessary to gather data from as wide a variety of laboratories as possible, in order to mitigate the risk that what appears to be an accurate algorithm may not have the broad applicability required of a clinical algorithm. No guidelines are yet available on the numbers of annotations, images and laboratories that are needed to capture the variation that is seen in the real world, and statistical studies will be needed for application to properly determine this.

Given the inherent variation that exists in staining patterns from lab to lab, generalizing these algorithms will require a step change in the size and spread of samples from multiple laboratories. This is now driving the need for multinational data-lakes with large volumes of WSI in pathology and high quality annotations for AI training and validation from multiple laboratories. A number of initiatives are already underway to achieve this. In the UK, a large multi-million pound grant has been provided by the government Innovate UK programme to several clinical networks to support the construction of pathology data lakes for AI innovation. This is supported by a number of large industry partners to provide the infrastructure to support this initiative. This will provide a robust data environment for the development of reliable IVD-ready applications. Industry has to work within a very tightly regulated environment and satisfy regulatory authorities of efficacy and safety through comprehensive clinical studies, before releasing a product with clinical claims. While this is costly and time consuming, and will inevitably delay the introduction of computational pathology for clinical practice, it is a critical step and will ensure that AI applications undergo significant testing to ensure they are safe in the hand of professionals.

Finally, there is nervousness by some that AI will replace skills, resulting in fewer jobs for pathologists and this will drive resistance. While AI will inevitably result in the automation of some common tasks in diagnostic pathology, the vast majority of applications will benefit from combined human-machine intelligence. Pathologists are excellent at assessing tissue pathology in the context of multiple clinico-pathological data across a broad range of diseases—some of which occur together. AI currently works best in well-defined domains, but brings quantitative insights to that domain, overcoming the issues of standardization. Doing this automatically can increase the speed of tissue assessment and provide pathologists with critical data on the tissue patterns. It is this hybrid approach of computer-aided decision support that is likely to drive the adoption and success of AI where the pathologist and machine working in tandem bring the biggest benefits. In these settings, some have proposed that Intelligence Augmentation (IA) is a better term than AI to describe how computational pathology will drive improvements in diagnostic pathology.

## Conclusion

Computational pathology and the application of AI for tissue analytics is growing at a tremendous rate and has the potential to transform pathology with applications that accelerate workflow, improve diagnostics, and the clinical outcome of patients. There is a large gap between research studies and those necessary to deliver safe and reliable AI to the pathology community. As the demands of clinical AI become better understood, we will see this gap narrow. The field of pathology AI is still young and will continue to mature as researchers, clinicians, industry, regulatory organizations, and patient advocacy groups work together to innovate and deliver new technologies to health care providers: technologies which are better, faster, cheaper, more precise, and safe.

## Author Contributions

All authors have written various sections of this review. All have deep experience on the application of AI and deep learning in pathology applications working in the Philips image analysis hub.

### Conflict of Interest

The authors are employees of Royal Philips, Digital and Computational Pathology. The opinions expressed in this presentation are solely those of the author or presenters, and do not necessarily reflect those of Philips. The information presented herein is not specific to any product of Philips or their intended uses. The information contained herein does not constitute, and should not be construed as, any promotion of Philips products or company policies

## Footnotes

^1^TissueMark is not intended for diagnosis, monitoring or therapeutic purposes or in any other manner for regular medical practice. PathXL is the legal manufacturer and is a Philips company.
